# High Pressure Homogenization of Porcine Pepsin Protease: Effects on Enzyme Activity, Stability, Milk Coagulation Profile and Gel Development

**DOI:** 10.1371/journal.pone.0125061

**Published:** 2015-05-04

**Authors:** Bruno Ricardo de Castro Leite Júnior, Alline Artigiani Lima Tribst, Marcelo Cristianini

**Affiliations:** Department of Food Technology (DTA), School of Food Engineering (FEA), University of Campinas (UNICAMP), Monteiro Lobato, Campinas, S.P., Brazil; Agricultural University of Athens, GREECE

## Abstract

This study investigated the effect of high pressure homogenization (HPH) (up to 190 MPa) on porcine pepsin (proteolytic and milk-clotting activities), and the consequences of using the processed enzyme in milk coagulation and gel formation (rheological profile, proteolysis, syneresis, and microstructure). Although the proteolytic activity (PA) was not altered immediately after the HPH process, it reduced during enzyme storage, with a 5% decrease after 60 days of storage for samples obtained with the enzyme processed at 50, 100 and 150 MPa. HPH increased the milk-clotting activity (MCA) of the enzyme processed at 150 MPa, being 15% higher than the MCA of non-processed samples after 60 days of storage. The enzyme processed at 150 MPa produced faster aggregation and a more consistent milk gel (G’ value 92% higher after 90 minutes) when compared with the non-processed enzyme. In addition, the gels produced with the enzyme processed at 150 MPa showed greater syneresis after 40 minutes of coagulation (forming a more compact protein network) and lower porosity (evidenced by confocal microscopy). These effects on the milk gel can be associated with the increment in MCA and reduction in PA caused by the effects of HPH on pepsin during storage. According to the results, HPH stands out as a process capable of changing the proteolytic characteristics of porcine pepsin, with improvements on the milk coagulation step and gel characteristics. Therefore, the porcine pepsin submitted to HPH process can be a suitable alternative for the production of cheese.

## Introduction

Cheese production grows about 4% per year worldwide [1]. Traditionally, cheese is produced using calf rennet as the coagulant, but the tendency to reduce the early slaughter of steers due to their being low achievers in terms of meat production [2], has increasingly limited the use of calf rennet, resulting in an increasing search for enzymes to replace calf rennet.

A good coagulant replacer must have high specificity at the pH and temperature commonly used for cheesemaking [3, 4] and a low unspecific proteolysis, to avoid problems like reduction of manufacturing yield (due to loss of small peptides in the step of whey separation) and the appearance of defects on flavour (bitterness) and texture (brittle texture) [5]. The recombinant chymosin is considered a good replacer to calf rennet, due to its specific activity on κ-casein; however, it may be avoided in some markets due to its genetically modified organisms (GMO) origin. Therefore, the search for substitutes to chymosin produced by GMO is necessary.

Porcine pepsin, enzymatically classified as a pepsin B (EC 3.4.23.2), is a protease found in porcine stomachs. This enzyme has milk-clotting activity [6, 7], but its unspecific proteolytic activity hydrolyses bonds with Phe, Tyr, Leu or Val residues [8, 9], favouring the formation of undesirable peptides. Therefore, the application of this enzyme as a calf rennet substitute is rare [10].

High pressure homogenization (HPH), also known as dynamic high pressure (DHP), is a non-conventional process applied to fluid foods [11, 12]. Recently, some researchers have been dedicated to evaluating the effect of the process on enzymes, and have found cases in which HPH was able to improve [12, 13, 14], reduce [15, 16] or not alter [17] the activity and stability of enzymes [18]. The effects of HPH were dependent on the level of pressure homogenization applied, the temperature of the enzyme during the process, the nature of the enzyme studied and the pH of homogenization [19, 20, 21]. Specifically for milk coagulants, HPH was able to reduce the unspecific proteolytic activity of calf rennet, increase its specificity in the milk-clotting formation, improve enzyme stability with time and improve the characteristics of the milk gel produced [22, 23].

Therefore, considering the results obtained for general enzymes and specifically for calf rennet, it is to be expected that HPH would modify the activity of porcine pepsin protease, maybe improving its characteristics as a coagulant, which could open new markets for this enzyme in the cheesemaking industry. Thus, this study aimed to evaluate the influence of high pressure homogenization on the proteolytic characteristics, stability and coagulation profile of a commercial porcine pepsin protease.

## Materials and Methods

### Porcine pepsin protease and high pressure homogenization

A commercial porcine pepsin protease was used in the experiments (freeze dried powdered Porcine Pepsin) PEPSINA SUINA TS (Bela Vista, Santa Catarina, Brazil).

A Panda Plus High-Pressure Homogenizer (GEA-Niro-Soavi, Parma, Italy) was used in the assays. This equipment has a single acting intensifier pump that amplifies the hydraulic pressure up to 200 MPa and operates at a flow rate of 9 L.h^-1^.

### Effect of high pressure homogenization on a commercial porcine pepsin protease

#### High pressure homogenization processing

A volume of 2 L of the porcine pepsin solution was prepared at 0.1% (w/v) in 0.2 M sodium acetate buffer (pH 5.6) and homogenized under pressures of 0, 50, 100, 150 and 190 MPa, using an inlet temperature of 23°C. A non-processed sample of porcine pepsin protease was evaluated as the control sample. The differences between the non-processed (control) and the sample processed at 0 MPa lays on the fact that the latter was pumped through the equipment valve (in a narrow gap) without pressure application, while the sample non-processed (control) was not subjected to the HPH process in any condition. Samples (200 mL) were collected and cooled to 23°C, and the temperatures were measured using a digital T type thermocouple (Multithermometer (Brazil)). The maximum pressure levels were chosen considering the operational capacity of the equipment [22].

The relative proteolytic activity (RPA) and relative milk-clotting activity (RMCA) were determined immediately after the end of processing (time 0h) and after 7, 14, 30 and 60 days. The rheological assays were carried out at time 0h and after 60 days. The samples were stored under refrigeration (4°C) throughout the period.

#### Relative proteolytic activity (RPA) determination

The proteolytic activity of porcine pepsin protease was determined according to the method described in [24], using an enzyme solution at a concentration of 0.1% w/v and sodium caseinate at 0.5% w/v (Sigma Aldrich, USA) as the substrate.

The enzymatic activity was calculated according to Eq 1 [24].

$$\frac{\text{U}}{\text{mL}}=\left({\Delta \text{Abs}}_{280\;\text{nm}}\cdot 10\cdot \text{dilution factor}\right)/\left(0.6\cdot 40\right)$$ (Eq. 1)

Where Abs_280nm_ is the absorbance value measured at 280 nm, 10 is the factor for converting absorbance into activity (considering that 1 U is determined by the variation of 0.1 on sample absorbance), the dilution factor is the percentage of enzyme dissolved in the solution, 0.6 is the enzyme solution volume added (milliliters) and 40 is the reaction time (minutes).

The relative proteolytic activity (RPA) was calculated considering the activity of the HPH and non-processed samples according to Eq 2 [22]:

$$\text{RPA}=\left(\frac{{\text{enzyme activity}}_{{\text{after}}_{{\text{HPH}}_{{\text{and}}_{{\text{or}}_{\text{storage}}}}}}}{{\text{enzyme activity}}_{\text{non}-{\text{processed}}_{{\text{sample}}_{{\text{at}}_{0\text{h}}}}}}\right)\cdot 100$$ (Eq. 2)

#### Relative milk-clotting activity (RMCA) determination

The milk-clotting activity was determined according to the method described in [24], using skimmed milk powder reconstituted at 10% (w/v) (pH 6.65, 3.2% protein, 9.2% non-fat solids, Tagará Foods, Brazil) with the addition of a 0.01 M CaCl_2_ and the enzyme solution at a concentration of 0.00015%, w/v. Clot formation was determined by manual tube rotation and the time taken for the first particles to form noted. One milk-clotting activity unit (MCA) was defined as the amount of enzyme required to clot 1 mL of substrate in 40 min at 35°C. The MCA was calculated using Eq 3 [24]:

$$\text{Units of MCA}=\left(\frac{2400}{\text{t}}\right)\cdot \left(\frac{\text{S}}{\text{E}}\right)$$ (Eq. 3)

Where *t* is the time (seconds) necessary for clot formation, S is the milk volume (5 mL) and E the enzyme solution volume (0.5 mL).

The RMCA was calculated considering the MCA of the HPH processed and non-processed samples, according to Eq 4 [22]:

$$\text{RMCA}=\left(\frac{{\text{MCA}}_{{\text{after}}_{{\text{HPH}}_{{\text{and}}_{{\text{or}}_{\text{storage}}}}}}}{{\text{MCA}}_{\text{non}-{\text{processed}}_{{\text{sample}}_{{\text{at}}_{0\text{h}}}}}}\right)\cdot 100$$ (Eq. 4)

#### Rheological assays

Milk coagulation was evaluated using a time sweep low deformation oscillatory test in a rheometer with controlled stress (AR2000ex, TA Instruments, USA) for up to 90 minutes. These assays were carried out using 60 mL of skimmed milk powder reconstituted at 10% (w/v) with the addition of a 0.01 M CaCl_2_ solution. This mixture was pre-incubated at 35°C/ 10 min and subsequently 0.8 mL of enzyme solution (0.015%, w/v) added. The mixture was immediately transferred to the rheometer cup and the rheological study carried out by measuring the parameter G’ (storage modulus) at 3 min intervals for 90 min of the clotting process at 35°C according to the method described in [22].

### Characterization of the coagulation process and the gel formed using commercial porcine pepsin processed by high pressure homogenization

#### High pressure homogenization processing

A volume of 1 L of the commercial porcine pepsin solution prepared at 0.1% (w/v) in 0.2M sodium acetate buffer (pH 5.6) was homogenized at 150 MPa using an inlet temperature of 23°C. Samples (200 mL) were collected and cooled to 23°C and a non-processed sample of porcine pepsin protease evaluated as the control sample [23].

#### Capillary zone electrophoresis of the porcine pepsin-induced gels

The samples were first prepared using 60 mL of skim milk powder reconstituted at 10% (w/v) with the addition of 0.01 M CaCl_2_ and 0.05% (w/v) sodium azide (Merck, Darmstadt, Germany). The mixture was then pre-incubated at 35°C/ 10 minutes, and subsequently 360 μL of enzyme solution (0.1%, w/v) were added and the time count started. After 40 min, 3 h, 6 h, 18 h and 24 h of coagulation at 35°C, 20 mg samples were collected and prepared for Capillary zone electrophoresis (CZE) following the method described in [23]. Capillary zone electrophoresis (CZE) was then carried out using the methodology described in [23]. The capillary electropherogram separation of a 1:1:1 mixture of α_s_-CN, β-CN and κ-CN and identification of the major caseins by analysis of the casein standards are presented in [23].

#### Determination of spontaneous syneresis of the porcine pepsin-induced gels using the siphon method

An aliquot of 60 mL of skim milk powder reconstituted at 10% (w/v) with the addition of 0.01 M CaCl_2_ and 0.05% (w/v) sodium azide (Merck, Darmstadt, Germany) was pre-incubated at 35°C/ 10 minutes. Subsequently, 360 µL of enzyme solution (0.1%, w/v) were added and the time count started. After 40 min, 3 h, 6 h, 18 h and 24 h coagulation at 35 ºC, the samples were cooled to 5 ºC and spontaneous syneresis then measured and calculated using the siphon method described in [23].

#### Rheological assays of the coagulation process and porcine pepsin-induced gels

Milk coagulation and gel development were evaluated by monitoring the milk coagulation process using a time sweep in a low deformation oscillatory test in a controlled stress rheometer (AR2000ex, TA Instruments, USA) for up to 24 hours. These assays were carried out using 60 mL of skimmed milk powder reconstituted at 10% (w/v) with the addition of a 0.01 M CaCl_2_ solution. This mixture was pre-incubated at 35°C/ 10 min and subsequently, 360 µL of enzyme solution (0.1%, w/v) added. Immediately after enzyme addition, the sample was transferred to the rheometer cup and the parameters G' (storage modulus) and G” (loss modulus) were measured at 3 minute intervals (up to the first 40 minutes) and then at 10 minute intervals (up to 24 hours) of the clotting process at 35°C. The assay was performed following the methodology described in [23].

#### Determination of the three dimensional (3D) microstructure during the coagulation process and of the porcine pepsin-induced gels by confocal scanning laser microscopy (CSLM)

An aliquot of 1 mL of skim milk powder reconstituted at 10% (w/v) with the addition of 0.01 M CaCl_2_ and 0.05% (w/v) sodium azide (Merck, Darmstadt, Germany) was pre-incubated at 35°C/ 10 minutes. Subsequently, 6 µL of enzyme solution (0.1%, w/v) and 25 µL of fast-green FCF (0.1% w/v, in distilled water, Sigma–Aldrich, Ireland) (used to observe the protein matrix in a confocal microscope) were added and the time started. Immediately after the additions, 200 µL of the solution were transferred to an 8 chamber coverglass (a 10 mm deep cavity dish) and covered with a glass coverslip (0.17 mm thick) (Lab-Tek II Chambered Coverglass, USA) and confocal imaging carried out using the method described in [23]. The porosity, number of pores, average pore and total pore areas were determined by image analysis of the CLSM micrographs using image J software (Research Service Branch, National Institute of Health, Maryland, USA) equipped with “Pore Analysis” and “ComputeStats” plug-ins, according to the parameters and methodology cited in [23].

### Statistical analysis

The processes and analyses were carried out with three repetitions and each experimental unit was carried out in triplicate. The analysis of variance (ANOVA) was used to compare the effects of the different treatments and the Tukey test to determine the differences between them at a 95% confidence level. The statistical analyses were carried out using the STATISTICA 7.0 software–(StatiSoft, Inc., Tulsa, Okla., U.S.A.) and the results presented as the mean ± standard deviation.

## Results and Discussion

### Effect of high pressure homogenization on commercial porcine pepsin protease

#### Relative proteolytic activity (RPA) and relative milk-clotting activity (RMCA)

**Fig 1:**
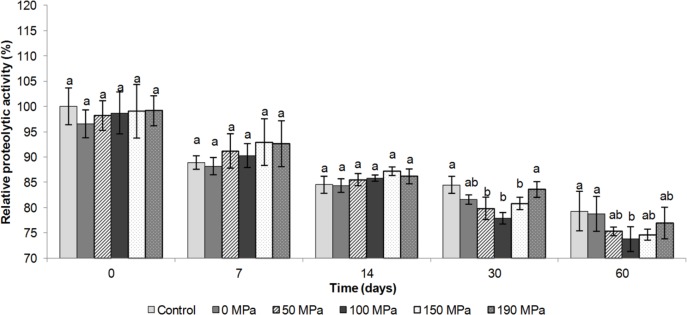
Relative proteolytic activity of the porcine pepsin subjected to the high pressure homogenization process. Different letters (a-b) mean significant difference evaluated by the Tukey test (p<0.05) between the non-processed and processed porcine pepsin samples. Evaluation is considered for each group of time (0, 7, 14, 30 and 60 days) separately.

The proteolytic activity measures the total ability of the enzyme to cleave casein in any fraction (α_s1_-, α_s2_-_,_ β-, ƙ-), forming peptides of different molecular weight. Therefore, proteolytic activity can be used as an indicative of the unspecific hydrolysis of the casein structure caused by an enzyme. Results showed that the proteolytic activity of porcine pepsin did not change (p>0.05) immediately after HPH and until the 14th day of storage, when compared with the activity measured for non-processed sample (Fig 1). Over this time, a continuous reduction in proteolytic activity was observed for all samples, reaching ~85% after 14 days. After 30 days of storage, the samples processed at 50 MPa, 100 MPa and 150 MPa showed lower activities (5 to 8% less) than the sample processed at 190 MPa or the non-processed sample (p<0.05) (Fig 1).

Similar behaviour was observed after 60 days of storage, where the sample processed at 100 MPa showed lower activity (~5% less) than the control sample or the sample processed at 0 MPa (p<0.05) (Fig 1). Thus the HPH process reduced the proteolytic activity of the porcine pepsin in solution after 30 days of storage. From the cheesemaking point of view, the reduction in excessive proteolytic activity could be a strategy for improving the cheese yield, flavour, and texture.

**Fig 2:**
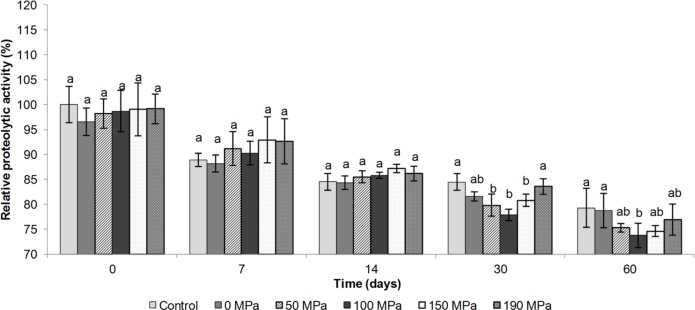
Relative milk-clotting activity of the porcine pepsin subjected to the high pressure homogenization process. Different letters (a-b) mean significant difference evaluated by the Tukey test (p<0.05) between the non-processed and processed porcine pepsin samples. Evaluation is considered for each group of time (0, 7, 14, 30 and 60 days) separately.

RMCA assess indirectly the specific activity of the enzyme on the hydrolysis of κ-casein at the linkage Phe_105_-Met_106_ [25] and the results of this experiment are shown in Fig 2. Immediately after processing, no significant differences were found for RMCA when compared with the control sample. From the 14th to 60th days of storage, the enzymes processed at 150 MPa and 100 MPa showed higher RMCA values than the non-processed enzyme or the enzyme processed at 190 MPa (p<0.05). After 60 days of storage, the sample processed at 150 MPa exhibited the highest MCA, which was 15% higher than the control. The HPH process at 150 MPa conferred greater stability on the milk-clotting activity of the enzyme, while HPH at 190 MPa just reduced the RMCA, thus the effect of HPH on RMCA is pressure-dependent.

The evaluation of the proteolytic and milk-clotting activities of the porcine pepsin after HPH processing indicated some most interesting results at 150 MPa and 100 MPa from the cheesemaking point of view. Although no differences were found immediately after homogenization, the enzyme processed at 150 MPa showed a potential improvement of its proteolytic profile during storage, due to a reduction in proteolytic activity and improvements in the milk-clotting activity.

The high pressure homogenization is a non-thermal processing that imparts to the processed fluid high turbulence, shear and cavitation that occurs as it flows through the homogenizer valve. This process is able to modify enzymes, causing increase or reduction on their activity, especially at non-optimum conditions [14, 21], and increase their stability [20]. The HPH effects on enzymes depend on the enzyme type, concentration, processing temperature, number of cycles [18] being difficult to predict results based on previous results.

Scarce data about HPH on milk coagulant have been published. Previous results showed a reduction of 52% in the proteolytic activity of calf rennet (enzyme extracted from calf abomasum containing 94% of chymosin and 6% of pepsin) after process at 190 MPa [22]. By comparing this result with the obtained in the present work, it was concluded that the porcine pepsin has greater resistance to HPH than calf rennet, which might indicate that pepsin is less susceptible to HPH effects than chymosin.

The enzyme activity modifications by HPH have been linked to conformational alterations caused by this process on the quaternary, tertiary and secondary structures [13]. These effects are mainly observed due to exposure of sulfhydryl and hydrophobic groups and residues of tyrosine and tryptophan [20, 26].

#### Rheological evaluation

**Fig 3:**
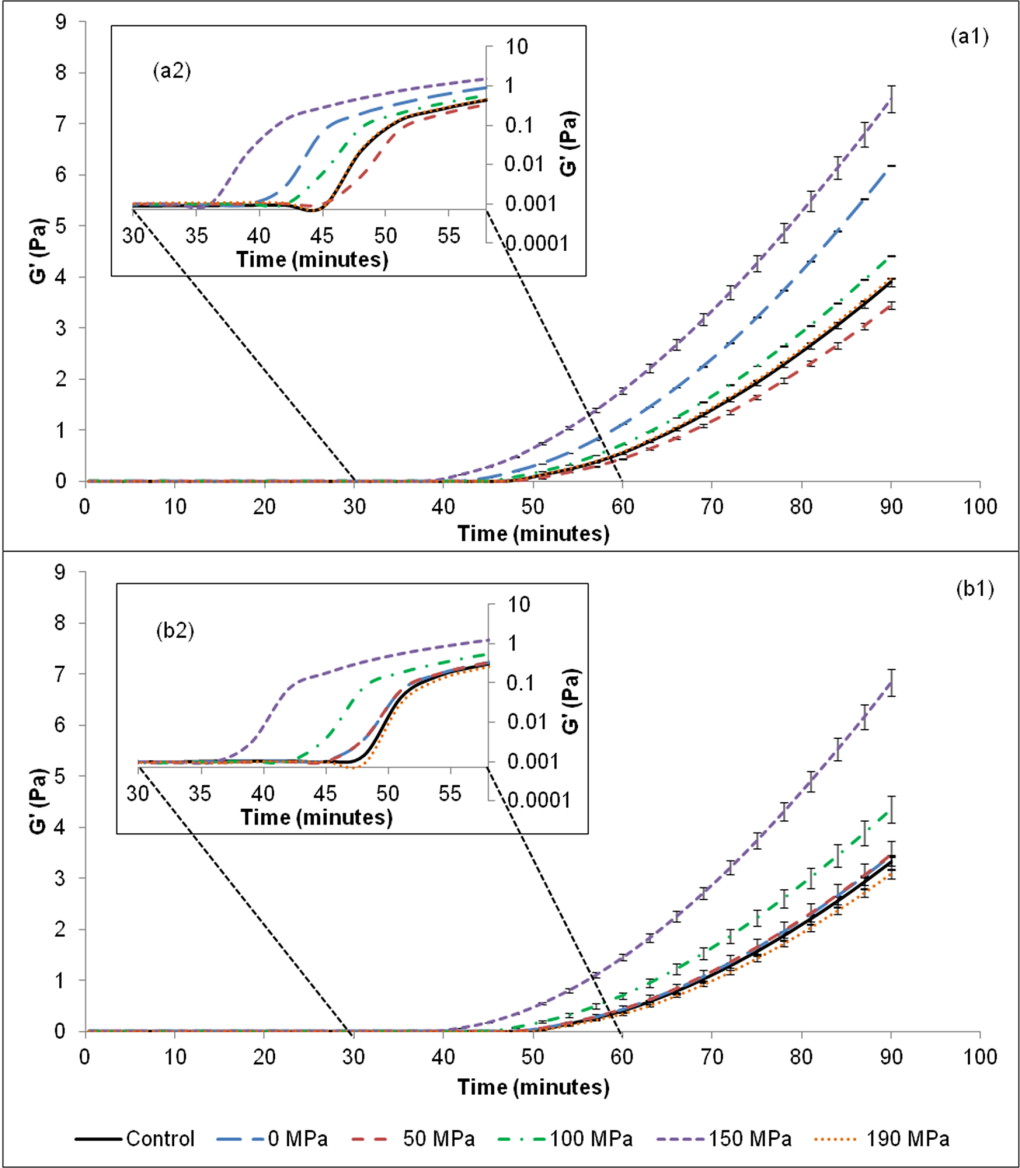
Evaluation of milk coagulation subjected to porcine pepsin processed by to high pressure homogenization at different storage time: (a1) immediately after processing (0h) (a2) log scale, and (b1) after 60 days storage at 4°C (b2) log scale. ** Values are means of replicates (n = 6).

The storage modulus (G') describes the elastic (solid) behaviour of the sample and can represent the milk coagulation phenomenon (Fig 3) [22]. Early aggregation can be better visualized using a log scale (Fig 3A2 and 3B2). Aggregation was observed after 36 minutes for the sample that used porcine pepsin homogenized at 150 MPa. In contrast, when the non-processed enzyme and the enzymes subjected to 50 and 190 MPa were used, aggregation only started after 45 minutes. Therefore using the enzyme processed at 150 MPa, coagulation occurred 20% faster than when using the non-processed enzyme. This may be due to the fast cleavage of the specific hydrophilic glycomacropeptide, preferably at the Phe_105_-Met_106_ site of κ-CN in the primary stage of coagulation. After 90 minutes of coagulation, the G’ values were 92% higher for the gel produced with porcine pepsin homogenized at 150 MPa than for the gels obtained using the non-processed enzyme (p<0.05). The differences found between the G’ values in the present study showed that an increment in pressure might cause changes in enzyme conformation, with consequent effects on coagulation. The differences between the results of non-processed (control) and sample processed at 0 MPa can be explained by the fact that when enzyme solution flows through the equipment it is subjected to a low shear due to equipment gap and, for this enzyme, this shear possibly was enough to cause some changes on enzyme activity. Similar results were obtained for α-amylase and β-galactosidase [16, 17].

The rheological results showed that HPH at 150 MPa positively affected the porcine pepsin, reducing the coagulation time and improving gel consistency.

Another rheological evaluation was carried out on the porcine pepsin in solution after 60 days of storage at 4°C (Fig 3B1), aiming to determine whether storage affected the coagulation profile of the enzymes in solution. The storage period led to a loss of milk-clotting activity for both the processed and non-processed enzymes, which was evidenced by the increase in time needed to start the aggregation process, corroborating with the RMCA results. Similar G’ values were found when comparing the results after 0 and 60 days of enzyme storage. Once again, coagulation using the enzyme processed at 150 MPa occurred faster than using the non-processed enzyme, since the time required to initiate the aggregation process was 25% lower for the 150 MPa-processed enzyme. After 90 minutes of coagulation, the G’ value was 106% higher for the enzyme processed at 150 MPa than for the non-processed enzymes. These results corroborate with the increase in enzyme stability in solution after the HPH process.

### Characterization of the coagulation process and gel formation using commercial porcine pepsin processed by high pressure homogenization

#### Proteolysis of porcine pepsin-induced gels as shown by capillary electrophoresis

**Fig 4:**
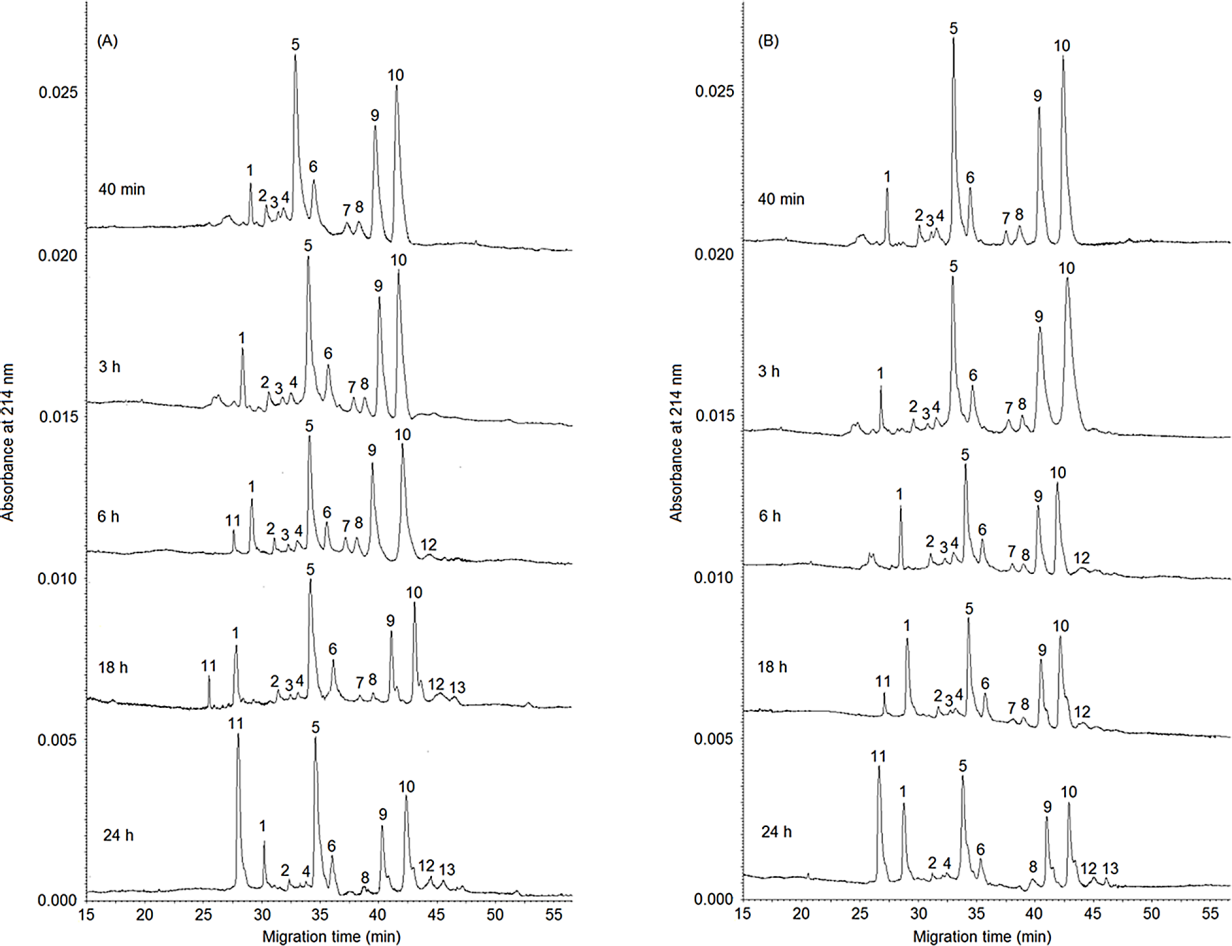
Capillary electrophoregram of the porcine pepsin-induced gels using (A) non-processed and (B) enzymes processed by high pressure homogenization at 150 MPa, throughout a 24 hour coagulation period at 35°C. Peak identification: 1: para-κ-CN; 2: α_s2_-CN; 3: α_s2_-CN; 4: α_s2_-CN (three phosphorylation states of the α_s2_-CN monomer were achieved); 5: α_s1_-CN; 6: α_s0_-CN (two phosphorylation states of the α_s1_-CN monomer were achieved); 7: κ-CN; 8: β-CN^B^; 9: β-CN^A1^; 10: β-CN^A2^ (two genetic variants of β-CN: A1 and A2); 11: γ_2_-CN; 12: αs_1_-I-CN 8P; 13: αs_1_-I-CN 9P.

Fig 4 shows the electropherograms of the porcine pepsin-induced gels during 24 hours at 35°C. The peaks are indicated on the electropherograms with serial numbers, and the respective identification is detailed in the figure caption.

When comparing the casein gels obtained with the enzymes subjected or not to high pressure homogenization, although the hydrolysis profiles were similar throughout the storage period, the non-processed enzyme caused slightly greater casein hydrolysis, especially of the β-CN fraction (peak 8: β-CN^B^; 9: β-CN^A1^; 10: β-CN^A2^, two genetic variants of β-CN: A1 and A2, [27, 28]) due to the higher specificity of pepsin for this fraction, with the formation of peak 11 after 6 h, identified as γ_2_-CN [29], and less hydrolysis of the αs_1_-CN 8P fraction (peak 5, [29]) forming peak 13, identified as the αs_1_-I-CN 8P fraction [29], after 18h. These peaks were only observed in the gel produced by the HPH enzyme after 18 h and 24 h, respectively. Fig 4 (A) shows that after 24h there was a delay of around 3 minutes for the appearance of the first peak (peak 11). This delay completely disappeared after peak 5.

The reduced proteolysis may have been due to the reduction of the proteolytic activity of the HPH processed porcine pepsin during storage (as observed in section 3.1.1, Fig 1). The hydrolysis profiles of κ-CN after 40 minutes and during gel storage were similar for the non-processed and processed enzymes. Thus the results evidenced the fact that the HPH process was able to reduce the unspecific proteolytic behaviour of the porcine pepsin after gel formation, with no effects on enzyme specificity.

The effect of HPH on porcine pepsin is important from an industrial point of view, since the reduction in the proteolytic profile might limit the considerable unspecific proteolysis during cheese ripening [23]. This undesirable proteolysis mainly affects the β-casein fraction, releasing bitter peptides that negatively affect the cheese flavour [30]. Additionally, the limitation of proteolysis avoids the cheese texture damage during its shelf life; besides, it ensures an adequate balance between intact proteins and peptides during cheese ripening [31]. Although a real improvement on cheese sensory quality is predictable after HPH process of porcine pepsin, no differences on nutritional characteristics are expected for cheese produced with non-processed and processed enzyme, since the coagulation time is not enough to cause excessive protein loss due to the intense proteolytic activity of the enzyme.

#### Spontaneous syneresis and rheological assays of the coagulation process and the development of the porcine pepsin-induced gels

**Fig 5:**
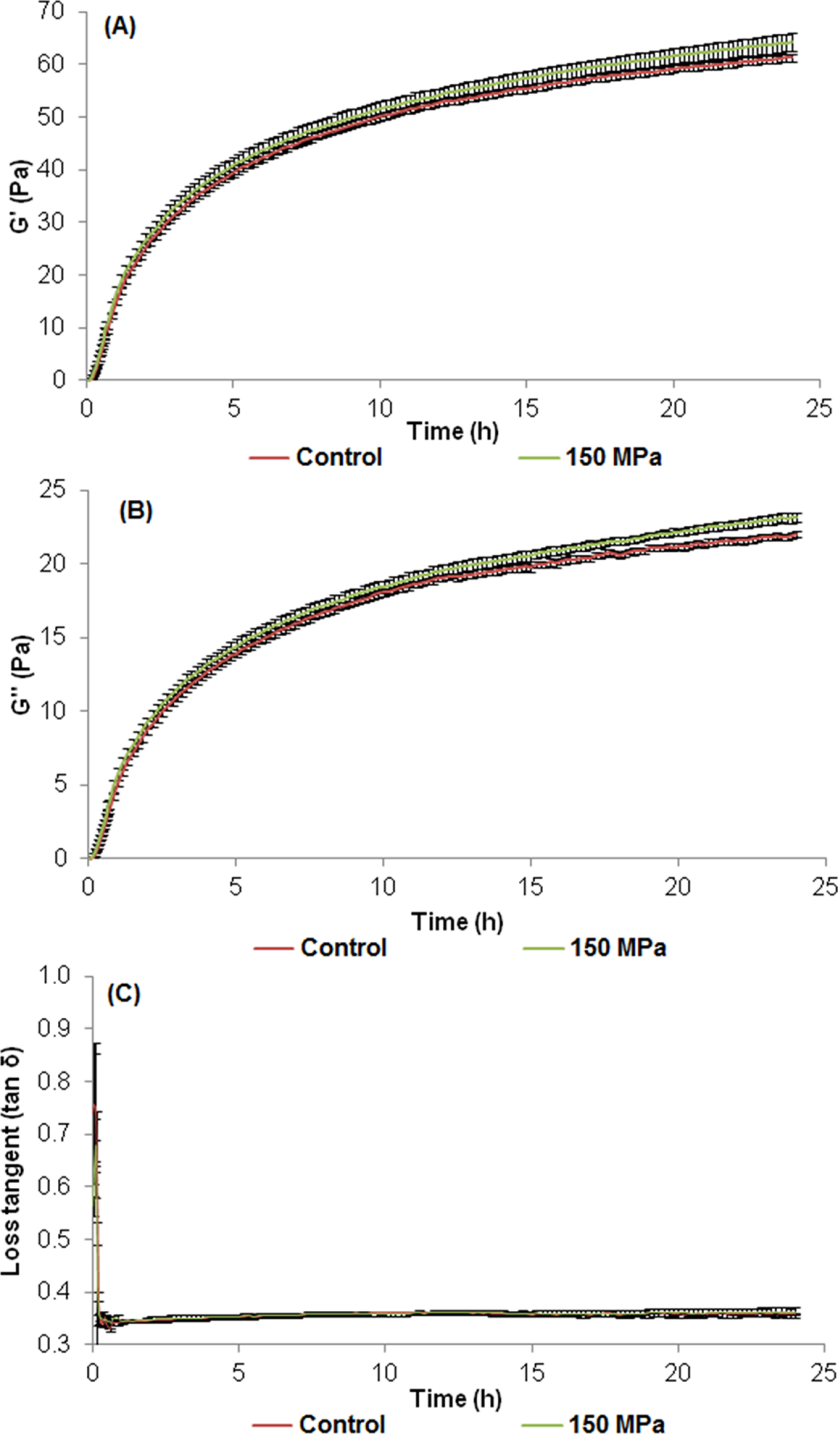
Evaluation of milk coagulation using a porcine pepsin enzyme subjected to high pressure homogenization (processed at 150 MPa—green lines) and a non-processed control (red lines) immediately after processing, throughout a 24 hours period at 35°C: (A) G’; (B) G”; (C) Loss tangent. ** Values are means of replicates (n = 6).

Syneresis of the porcine pepsin-induced gels produced with enzymes subjected or not to the HPH process was evaluated for 24 h at 35°C, and the results are shown in Table 1. Although after 40 minutes of coagulation the gels produced with the enzyme processed at 150 MPa showed significantly more syneresis than the gel produced with the non-processed enzyme (p<0.05), no differences were observed between the samples after 3 h (p>0.05). Curd syneresis naturally occurs due to the formation of new bonds between para-casein micelles, leading to compression of the casein micelles and whey expulsion [23, 32, 33, 34, 35]. The results showed that differences between the samples were only evident after 40 minutes of coagulation. Therefore, the change caused by HPH on the enzyme possibly affected its behaviour during milk coagulation (as observed in the rheological study—Figs 3 and 5), leading to faster aggregation, resulting in firmer gels and consequently greater syneresis.

**Table 1:** Rheological properties and whey exudation of milk gels produced using porcine pepsin processed by high pressure homogenization, throughout a 24 hour coagulation period at 35°C.

Coagulation time (h)	Whey exudation (%) **		Storage modulus		Loss modulus		Loss tangent (tan δ) *	
			G' (Pa) *		G'' (Pa) *			
	Control	150 MPa	Control	150 MPa	Control	150 MPa	Control	150 MPa
**0.33 (20 min)**	ND	ND	2.4 ± 0.3^b^ ^A^	3.2 ± 0.5 ^a^ ^A^	0.8 ± 0.1^b^ ^A^	1.1 ± 0.2^a^ ^A^	0.333±0.003^b^ ^A^	0.344±0.004^a^ ^A^
**0.67 (40 min)**	1.68 ± 0.17^b^ ^A^	2.33 ± 0.47^a^ ^A^	9.2 ± 0.4^b^ ^B^	10.4 ± 0.7^a^ ^B^	3.1 ± 0.1^b^ ^B^	3.6 ± 0.3^a^ ^B^	0.337±0.002^b^ ^A^	0.346±0.003^a^ ^A^
**3**	5.02 ± 0.84^a^ ^B^	5.38 ± 0.50^a^ ^B^	31.5 ± 0.7^a^ ^C^	32.8 ± 1.4^a^ ^C^	11.0 ± 0.3^a^ ^C^	11.5 ± 0.5^a^ ^C^	0.349±0.002^a^ ^B^	0.351±0.002^a^ ^B^
**6**	6.18 ± 0.79^a^ ^B^	6.84 ± 0.81^a^ ^C^	42.3 ± 0.8^a^ ^D^	43.7 ± 1.6^a^ ^D^	15.0 ± 0.3^a^ ^D^	15.5 ± 0.5^a^ ^D^	0.355±0.002^a^ ^C^	0.355±0.003^a^ ^B^ ^C^
**18**	11.05 ± 0.80^a^ ^C^	12.14 ± 1.33^a^ ^D^	57.8 ± 0.7^b^ ^E^	60.0 ± 1.4^a^ ^E^	20.6 ± 0.2^b^ ^E^	21.5 ± 0.5^a^ ^E^	0.356±0.003^a^ ^C^ ^D^	0.359±0.002^a^ ^C^ ^D^
**24**	14.82 ± 1.10^a^ ^D^	15.87 ± 1.96^a^ ^E^	61.3 ± 0.7^b^ ^F^	64.2 ± 1.7^a^ ^F^	22.0 ± 0.2^b^ ^F^	23.2 ± 0.5^a^ ^F^	0.359±0.003^a^ ^D^	0.361±0.002^a^ ^D^

* Means ± standard deviation of replicates (n = 6).

** Means ± standard deviation of replicates (n = 9).

a,b Significant differences evaluated by the Tukey test (p < 0.05) between control and 150 MPa-samples in the same row are indicated by different superscripts.

A-F Significant differences evaluated by the Tukey test (p < 0.05) between same sample over time in the same column are indicated by different superscripts.

ND: Not Determined.

Table 1 and Fig 5 show the results obtained for the rheological parameters of the milk gels (during 24h) produced using non-processed porcine pepsin and that processed at 150 MPa. The G’ and G” values (G” is a measure of the energy dissipated per cycle and describes the behavior of the sample as a viscous liquid [36]) showed increments up to 24 h, but the small differences between the results at 18 and 24 h showed few changes in the rate of milk clotting after 18 h, which became very slow. Higher G’ and G” values were obtained for the gels produced with HPH processed pepsin during the first 40 minutes, and after 18 and 24 hours of coagulation (p< 0.05).

These results demonstrated that the gels obtained with the HPH processed enzyme were initially more consistent and firmer, and the contraction of the casein aggregates up to 24 h increased gel consistence. The non-processed sample showed similar behaviour, but lower G’ and G” values were observed during the 24 hour period, possibly due to lower contraction of the network (less linkages of para-k-casein micelles) and greater unspecific proteolysis caused by the non-processed enzyme during storage. Therefore, it is possible to conclude that the HPH process of porcine pepsin resulted in more consistent gels with greater viscoelasticity after 24 h of coagulation, favouring the formation of firm, compact curds.

A tendency for greater losses of the tangent values (Table 1 and Fig 5) was observed for the gels prepared with the HPH processed enzyme, which was statistically significant after 3 hours of coagulation (*p*< 0.05). This finding corroborates the hypothesis of greater syneresis after gel formation due to greater contraction of the protein matrix, leading to the formation of a denser protein network.

Thus the results obtained for syneresis and rheology evidenced that the protein network was stronger from the beginning of coagulation to the end of porcine pepsin-induced gel development. The effects observed in these gels could possibly be of interest for cheese manufacture, by improving the cutting and mixing steps, leading to greater cheese yields (due to the increment in water binding capacity of the protein) and greater dry matter (due to reduced losses of small peptides to the whey) [23].

#### Microstructure of the coagulation of milk by porcine pepsin

**Fig 6:**
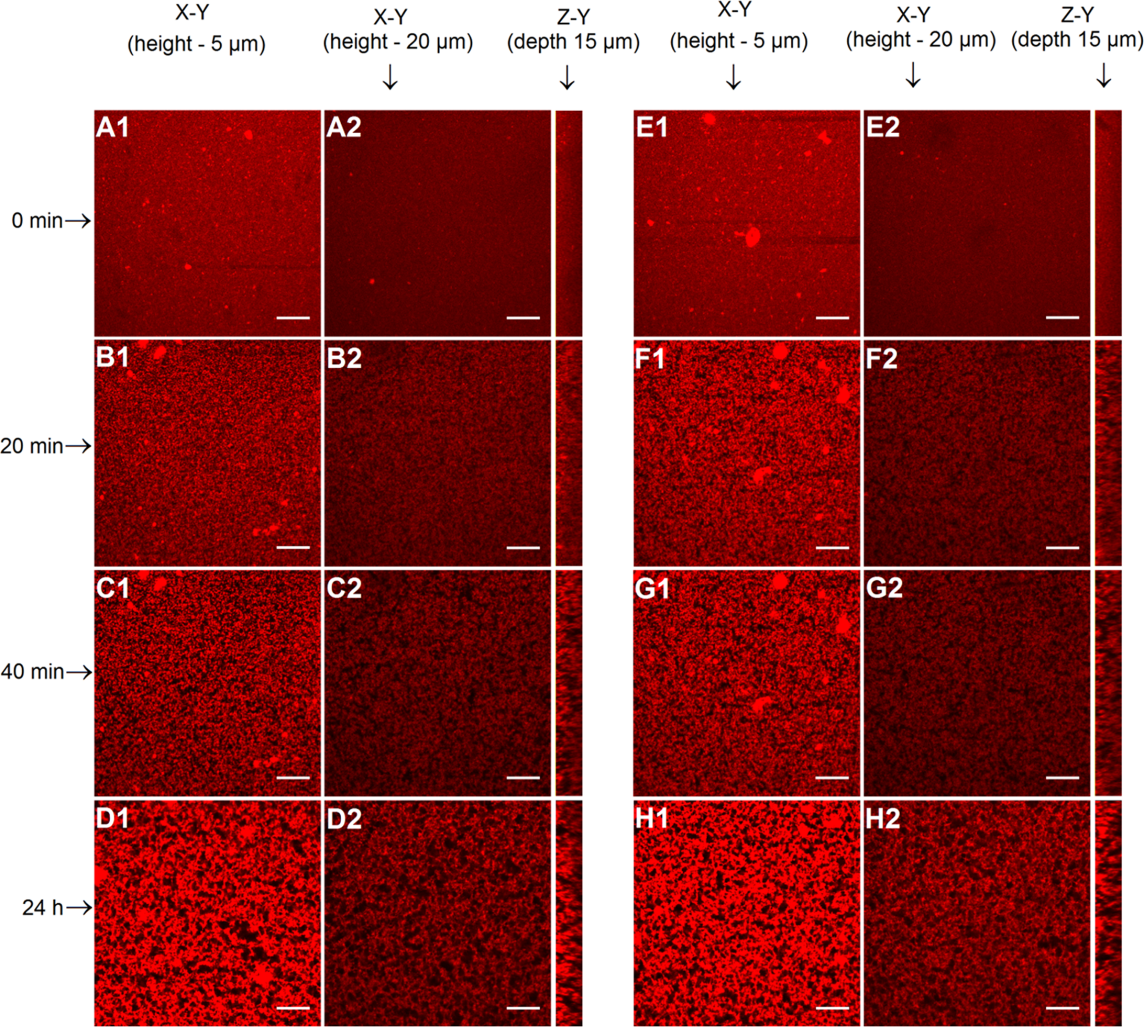
CLSM micrographs of gels obtained using a porcine pepsin enzyme processed at 150 MPa and non-processed porcine pepsin (A1-D1: control at a height of 5 μm; A2-D2: control at a height of 20 μm; E1-H1: 150 MPa at a height of 5 μm; E2-H2: 150 MPa at a height of 20 μm) throughout a 24 hour period at 35°C. The fast-green FCF stained protein appears red and the serum phase appears black in these images. Each set of images is presented with two views: the X–Y (a height of 5 and 20 **μ**m from the bottom—the surface of the coverglass) and the Z–Y (right) projections. For each sample, 20 adjacent planes were acquired with the separation between the planes kept constant at 0.75 **μ**m, giving a total observation depth of 15 **μ**m. The scale bars are 20 **μ**m in length.

**Fig 7:**
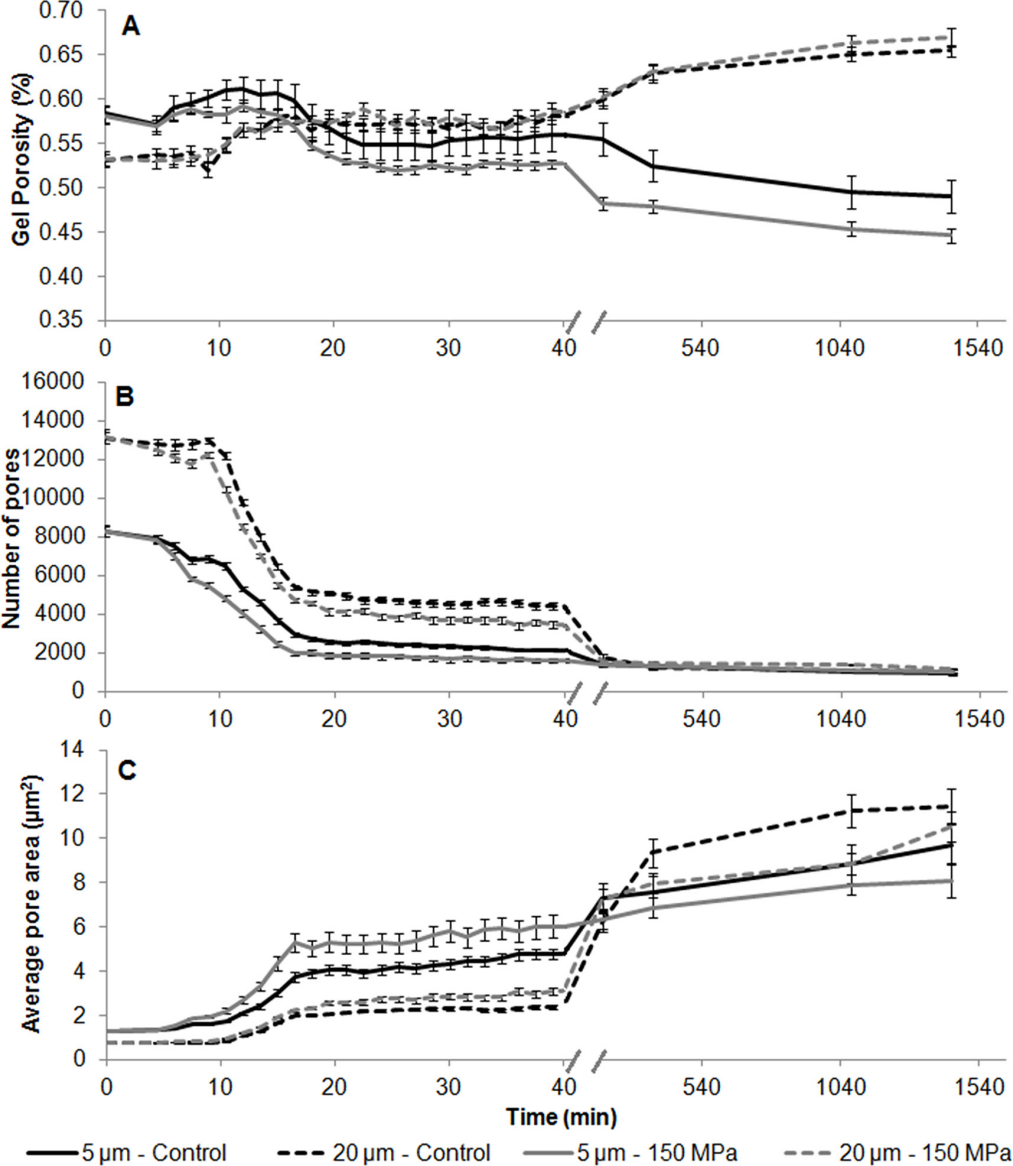
Gel porosity (A), number of pores (B) and average pore area (C) of gels obtained using porcine pepsin enzyme processed at 150 MPa and non-processed porcine pepsin. The results are expressed as the mean ± standard deviation (n = 6).

Fig 6 shows the images obtained by confocal scanning laser microscopy (CSLM) of the enzymatic coagulation of milk and the formation of a gel using the enzyme subjected or not to 150 MPa. The gel porosity, total number of pores and average pore area are shown in Fig 7 and Table 2. The intervals of the Table 2 were chosen considering the beginning of the aggregation, approximately half coagulation time (~ 20 min), end of the aggregation (end of the second phase of the coagulation, 40 min) and final of the development of the gel (24 h).

**Table 2:** Gel porosity, average pore area and number of pores of gels obtained using porcine pepsin processed at 150 MPa and non-processed porcine pepsin.

		**Sample height**			
		**5 μm**			
	Sample	0 min (Blank)	20 min	40 min	24 h
Gel Porosity (%)*	Control	0.5834 ± 0.0104 ^a^ ^A^	0.5686 ± 0.0191 ^a^ ^B^	0.5606 ± 0.0188 ^a^ ^B^	0.4910 ± 0.0186 ^a^ ^C^
	150 MPa	0.5814 ± 0.0095 ^a^ ^A^	0.5380 ± 0.0037 ^b^ ^B^	0.5269 ± 0.0054 ^b^ ^C^	0.4457 ± 0.0079 ^b^ ^D^
Number of pores*	Control	8254 ± 271 ^a^ ^A^	2567 ± 101 ^a^ ^B^	2139 ± 76 ^a^ ^C^	925 ± 71 ^a^ ^D^
	150 MPa	8300 ± 273 ^a^ ^A^	1852 ± 139 ^b^ ^B^	1601 ± 123 ^b^ ^B^	1009 ± 85 ^a^ ^C^
**Average pore area (μm** ^**2**^ ) *	Control	1.28 ± 0.03 ^a^ ^A^	4.04 ± 0.23 ^a^ ^B^	4.78 ± 0.23 ^a^ ^C^	9.71 ± 0.89 ^a^ ^D^
	150 MPa	1.28 ± 0.02 ^a^ ^A^	5.31 ± 0.43 ^b^ ^B^	6.02 ± 0.51 ^b^ ^B^	8.09 ± 0.78 ^b^ ^C^
		**Sample height**			
		**20 µm**			
	Sample	0 min (Blank)	20 min	40 min	24 h
Gel Porosity (%)*	Control	0.5315 ± 0.0057 ^a^ ^A^	0.5739 ± 0.0098 ^a^ ^B^	0.5804 ± 0.0088 ^a^ ^B^	0.6546 ± 0.0067 ^a^ ^C^
	150 MPa	0.5323 ± 0.0095 ^a^ ^A^	0.5731 ± 0.0052 ^a^ ^B^	0.5864 ± 0.0094 ^a^ ^B^	0.6698 ± 0.0099 ^b^ ^C^
Number of pores*	Control	13094 ± 291 ^a^ ^A^	5086 ± 122 ^a^ ^B^	4432 ± 198 ^a^ ^C^	1044 ± 77 ^a^ ^D^
	150 MPa	13180 ± 359 ^a^ ^A^	4107 ± 160 ^b^ ^B^	3447 ± 185 ^b^ ^C^	1162 ± 66 ^a^ ^D^
**Average pore area (μm** ^**2**^ ) *	Control	0.74 ± 0.03 ^a^ ^A^	2.06 ± 0.05 ^a^ ^B^	2.39 ± 0.09 ^a^ ^C^	11.46 ± 0.77 ^a^ ^D^
	150 MPa	0.73 ± 0.01 ^a^ ^A^	2.54 ± 0.10 ^b^ ^B^	3.10 ± 0.17 ^b^ ^C^	10.53 ± 0.69 ^a^ ^D^

* Means ± standard deviation of replicates (n = 6).

a,b Significant differences evaluated by the Tukey test (p < 0.05) between control and 150 MPa-samples in the same row are indicated by different superscripts.

A-D Significant differences evaluated by the Tukey test (p < 0.05) between same sample over time in the same column are indicated by different superscripts.

These images elucidate the effect of HPH on the behaviour of the porcine pepsin during gel formation. The images clearly show that, 20 minutes after enzyme addition, protein aggregation was faster for gels produced with the HPH processed enzyme (150 MPa). At the end of the coagulation step (40 minutes), similar micrographs were observed for all gels. After 24 h, a more compact protein network was observed for the gel obtained with the processed enzyme, corroborating the results obtained in rheology.

The gel porosity at 5 μm from the bottom of the sample was reduced due to aggregation and sedimentation of the protein network. To the contrary, the porosity at 20 μm increased due excessive whey expulsion from the protein matrix. Comparing the results obtained for each enzyme it was observed that, from 20 minutes to 24 hours of coagulation, the porosity at 5 μm was lower for gels produced with high pressure homogenized enzymes (*p*<0.05, Table 2 and Fig 7). In contrast, at 20 μm, the gel porosity was only different after 24 hours of coagulation, the gel produced with the processed enzyme being more porous (*p*<0.05), which possibly is related to the reduction of unspecific activity caused by HPH on the porcine pepsin protease, resulting in less formation of small peptides during this 24h. These peptides, possibly formed in greater amount for non-processed sample, probably are the responsible for the lower porosity at 20 μm observed for the gel produced with this enzyme.

In addition, at 5 μm, reductions in the total number of pores and increases in the average pore area were observed with time, which is directly linked to protein aggregation. The gel obtained with the HPH processed enzyme showed a faster reduction in total pores with increasing pore area, presenting a significant difference (up to 40 minutes of coagulation) when compared with the gel produced using the non-processed enzyme (p<0.05). From 40 minutes to 24 hours, the opposite behaviour was observed for the gels produced with the processed and non-processed enzymes, with larger numbers of pores and smaller areas for the gel produced with the HPH processed enzyme. On the other hand, for non-processed sample, a continuous increase of the average pore size was observed at 5 μm, which can be linked to its high unspecific activity that hydrolyzes the milk protein with consequent increase of pore sizes over time.

At a distance of 20 μm, the results also showed a reduction in the total number of pores and an increase in the average pore area with coagulation time. Once again the gel obtained with the HPH processed enzyme showed a faster reduction of total pores with increasing pore area during the first 40 minutes. From 40 minutes to 24 hours, similar behaviour was observed, with a greater number of pores and reduced average pore area for the gel produced with the processed enzyme. This phenomenon could possibly be attributed to the greater number of para-κ-casein linkages in the milk coagulation step, resulting in greater compaction and sedimentation of the protein network and maintenance of the links between the casein micelles, due to reduced gel proteolysis by the processed enzyme with time.

The comparison between these results with the obtained to milk gels produced by calf rennet (processed or not by HPH) shows that there is a difference in the average size of the pore measured at 5 μm. For gels produced with calf rennet (HPH processed or not) it was observed a reduction in the pore average size from 40 minutes to 24h, following a similar rate for gels produced with processed and non-processed enzyme (which might be linked to the initial low proteolytic profile of the non-processed sample). On the other hand, samples produced with porcine pepsin showed a maintenance (HPH processed enzyme) or increment (non-processed enzyme) of the average size of the pore. This difference can be explained by the pepsin having a higher unspecific activity than calf rennet and this residual activity possibly does not allow the reduction of pores over time. For gels produced with HPH pepsin, it was observed maintenance of the pore average size while for non-processed sample, an increase in the pore size was observed.

## Conclusions

High pressure homogenization did not affect the proteolytic and milk clotting activities of porcine pepsin immediately after processing. However, during storage, a reduction in proteolytic activity and an increase in milk-clotting activity were observed, especially for the enzyme subjected to 150 MPa. In addition, the gel produced with the enzyme processed at 150 MPa presented a 20% faster start, and was more consistent (G’ value 92% higher at 90 minutes immediately after processing) than the gel obtained with the non-processed enzyme. The milk gel produced with porcine pepsin processed at 150 MPa was more compact, firmer and less porous, and promoted more whey exudation from the protein matrix when compared with the gel obtained using the non-processed enzyme. After 24 h of coagulation, the gel produced using the high pressure homogenized enzyme maintained its consistency due to its lower proteolytic profile and greater milk-clotting activity.

Thus HPH could be applied as a method to improve the hydrolytic characteristics of pepsin porcine, possible allowing the use of porcine pepsin as an alternative coagulant for the cheesemaking industry. The choice of this enzyme instead of other coagulants (recombinant chymosin, calf of adult bovine rennet) will be based on the price of enzyme, hydrolytic profile and characteristics desired for the cheese.
